# Unfolding Simulations Reveal the Mechanism of Extreme Unfolding Cooperativity in the Kinetically Stable α-Lytic Protease

**DOI:** 10.1371/journal.pcbi.1000689

**Published:** 2010-02-26

**Authors:** Neema L. Salimi, Bosco Ho, David A. Agard

**Affiliations:** 1 Graduate Group in Biophysics, University of California San Francisco, San Francisco, California, United States of America; 2 Howard Hughes Medical Institute, University of California San Francisco, San Francisco, California, United States of America; 3 Department of Biochemistry and Biophysics, University of California San Francisco, San Francisco, California, United States of America; University of Houston, United States of America

## Abstract

Kinetically stable proteins, those whose stability is derived from their slow unfolding kinetics and not thermodynamics, are examples of evolution's best attempts at suppressing unfolding. Especially in highly proteolytic environments, both partially and fully unfolded proteins face potential inactivation through degradation and/or aggregation, hence, slowing unfolding can greatly extend a protein's functional lifetime. The prokaryotic serine protease α-lytic protease (αLP) has done just that, as its unfolding is both very slow (t_1/2_ ∼1 year) and so cooperative that partial unfolding is negligible, providing a functional advantage over its thermodynamically stable homologs, such as trypsin. Previous studies have identified regions of the domain interface as critical to αLP unfolding, though a complete description of the unfolding pathway is missing. In order to identify the αLP unfolding pathway and the mechanism for its extreme cooperativity, we performed high temperature molecular dynamics unfolding simulations of both αLP and trypsin. The simulated αLP unfolding pathway produces a robust transition state ensemble consistent with prior biochemical experiments and clearly shows that unfolding proceeds through a preferential disruption of the domain interface. Through a novel method of calculating unfolding cooperativity, we show that αLP unfolds extremely cooperatively while trypsin unfolds gradually. Finally, by examining the behavior of both domain interfaces, we propose a model for the differential unfolding cooperativity of αLP and trypsin involving three key regions that differ between the kinetically stable and thermodynamically stable classes of serine proteases.

## Author Summary

Proteins, synthesized as linear polymers of amino acids, fold up into compact native states, burying their hydrophobic amino acids into their interiors. Protein folding minimizes the non-specific interactions that unfolded protein chains can make, which include aggregation with other proteins and degradation by proteases. Unfortunately, even in the native state, proteins can partially unfold, opening up regions of their structure and making these adverse events possible. Some proteins, particularly those in harsh environments full of proteases, have evolved to virtually eliminate partial unfolding, significantly reducing their rate of degradation. This elimination of partial unfolding is termed “cooperative,” because unfolding is an all-or-none process. One class of proteins has diverged into two families, one bacterial and highly cooperative and the other animal and non-cooperative. We have used detailed simulations of unfolding for members of each family, α-lytic protease (bacterial) and trypsin (animal) to understand the unfolding pathways of each and the mechanism for the differential unfolding cooperativity. Our results explain prior biochemical experiments, reproduce the large difference in unfolding cooperativity between the families, and point to the interface between α-lytic protease's two domains as essential to establishing unfolding cooperativity. As seen in an unrelated protein family, generation of a cooperative domain interface may be a common evolutionary response for ensuring the highest protein stability.

## Introduction

α-lytic protease (αLP), a prokaryotic serine protease of the chymotrypsin family, has evolved an unusual energetic landscape, providing it a functional advantage over its metazoan homologs. Unlike most proteins, αLP's active state is not stabilized by thermodynamics, but by a large kinetic barrier to unfolding, with an unfolding t_1/2_ of ∼1 year.[1] While thermodynamically stable homologs like trypsin have similar unfolding rates, they are degraded at rates up to 100x faster than αLP under highly proteolytic conditions.[2],[3] In addition, the rates of αLP unfolding and degradation are nearly identical, indicating that partial unfolding leading to proteolysis is negligible. Therefore, αLP's functional advantage is derived from not only its very slow unfolding, which it shares with trypsin, but also its suppression of local unfolding events that would render it protease-accessible. Thus, it appears that the evolution of αLP has generated such extreme cooperativity in unfolding in order to maximize its functional lifetime under harsh conditions. The cost of maximizing resistance to unfolding comes in the form of extremely slow folding (t_1/2_ ∼1800 years) and the consequent loss of thermodynamic stability of the active state relative to the unfolded state.[1],[3] However, αLP also evolved a large Pro-region folding catalyst, which speeds folding by nine orders of magnitude and is then degraded by the mature protease, decoupling the folding and unfolding landscapes so that unfolding resistance can be maximized.[1],[2],[4]

**Figure 1:**
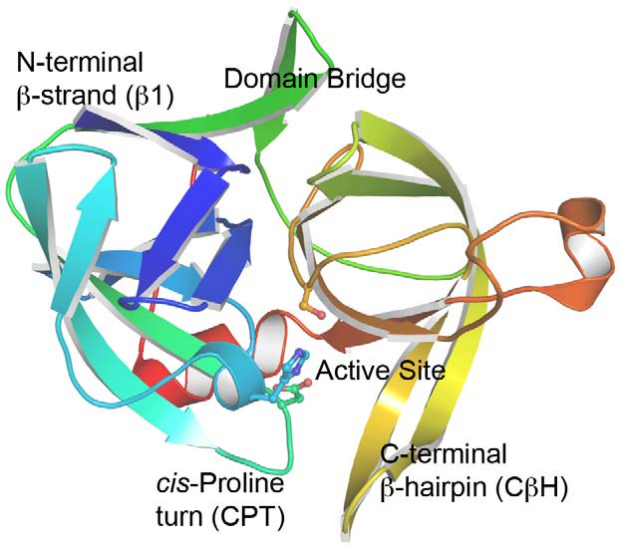
The structure of αLP. The molecule is colored dark blue at the N-terminus progressing to red at the C-terminus. Important structural regions for this work are labeled, including the active site (the catalytic triad of H57, D102, and S195 are represented in ball-and-stick), the N-terminal β-strand (β1, blue), the *cis*-proline turn (CPT, teal), the Domain Bridge (green), and the C-terminal β-hairpin (CβH, yellow).

Given αLP's unusual energetic landscape and its reliance on kinetic stability, much effort has focused on elucidating its unfolding mechanism in detail. Native-state hydrogen-deuterium exchange showed over half of its 194 backbone amides are well-protected from exchange, and 31 have protection factors greater than 10^9^.[2] This extreme rigidity is spread throughout both domains and is indicative of αLP's high unfolding cooperativity. Thermodynamic decomposition of the unfolding energetics into entropic and enthalpic contributions suggested a prominent role for the extensive domain interface in unfolding, with the critical step involving solvation of the domain interface while the individual domains remain relatively intact.[5] Mutational studies on αLP inspired by the acid-resistant homolog NAPase were consistent with this hypothesis. The distribution of salt-bridges in NAPase and αLP differ markedly; replacement of a salt-bridge at αLP's domain interface with an intra-domain salt-bridge (as in NAPase) resulted in significant increases in αLP's resistance to low pH unfolding.[6] A major component of the domain interface, the Domain Bridge (Figure 1), is the only covalent linkage between the two domains. This structure exists only in prokaryotic proteases and varies considerably among αLP and its homologs. The area buried by the domain bridge is inversely correlated with the high-temperature unfolding rate for four kinetically stable proteases, indicating both its relevance and that it is weakened early in unfolding.[7] Another domain interface component is a β-hairpin in the C-terminal domain (CβH), unique to kinetically stable proteases, that forms part of the active site (Figure 1). Substitution of a more stable β-turn was consistent with an unfolding pathway where CβH loses its domain interface contacts early in unfolding.[8] Despite much progress, we still lack a global picture of αLP unfolding, especially at high resolution.

For higher-resolution views of protein folding/unfolding, researchers have often turned to ϕ-value analysis.[9]–[12] These studies involve large-scale protein engineering experiments which investigate the molecule's folding and unfolding kinetics after making perturbing mutations, normally hydrophobic deletions. By analyzing sufficiently large numbers of perturbations, structure in the transition state ensemble (TSE) can be inferred and a folding/unfolding mechanism can be proposed. Unfortunately, the extremely slow folding and unfolding rates for αLP make large-scale ϕ-value analysis on αLP impractical. As an alternative, we decided to investigate the αLP unfolding pathway computationally in order to explain previous experiments and guide new ones.

High-temperature molecular dynamics (MD) unfolding simulations offer the highest structural and temporal resolution for studying protein unfolding, but their results must be validated experimentally. Since unfolding rates for proteins are typically very slow under physiological conditions (ranging from minutes to a year for proteins such as αLP), very high temperatures (450–500 K) are required to accelerate the unfolding into the ns range required for computational analysis. As a consequence, initially there was significant concern as to the relevance of the high temperature TSEs to real proteins under physiological conditions. Daggett and co-workers have been pioneers in this field, using Chymotrypsin Inhibitor 2 (CI2) as a model system and have shown that the simulated unfolding calculations agree remarkably well with experimental ϕ-values and were even able to predict faster folding mutants.[13]–[16] Further work on other proteins by multiple groups has established MD unfolding simulations as a useful tool in examining protein unfolding at atomic resolution while correlating well with experiments.[17]–[20]

A critical step in analyzing unfolding simulations is accurately pinpointing the TSE from the multitude of conformations generated. Because the TSE is experimentally accessible through a molecule's folding and unfolding kinetics, its identification computationally can be used for both explanatory and predictive purposes. Various methods for identifying the TSE have been used in the past, breaking down into conformational clustering and landscape methods.[13], [15], [17], [19], [21]–[23] Conformational clustering relies on all-versus-all comparisons of conformations, often by Cα RMSD, while landscapes separating native from unfolded structures can be generated using properties of the conformations, such as the fraction of native contacts or secondary structure.

Here, we report the results of multiple MD simulations carried out at high temperature in order to probe the mechanism of αLP's extremely cooperative unfolding. Due to the robustness and cooperativity of αLP unfolding, the same TSE is obtained using either conformational clustering or landscape methods. The simulated unfolding pathway for αLP matches well with previously described experiments and provides atomic resolution to previous models for αLP unfolding which highlight the role of the domain interface. In addition, we have performed similar simulations on trypsin with the goal of understanding the observed experimental differences in unfolding cooperativity. Through a novel method for calculating cooperativity in MD simulations, we show αLP unfolds significantly more cooperatively than trypsin, mirroring the experimental results. Finally, by analyzing the domain interfaces of both proteins during unfolding, we propose a mechanism for how this differential cooperativity is achieved.

## Results

### Unfolding Simulations

**Figure 2:**
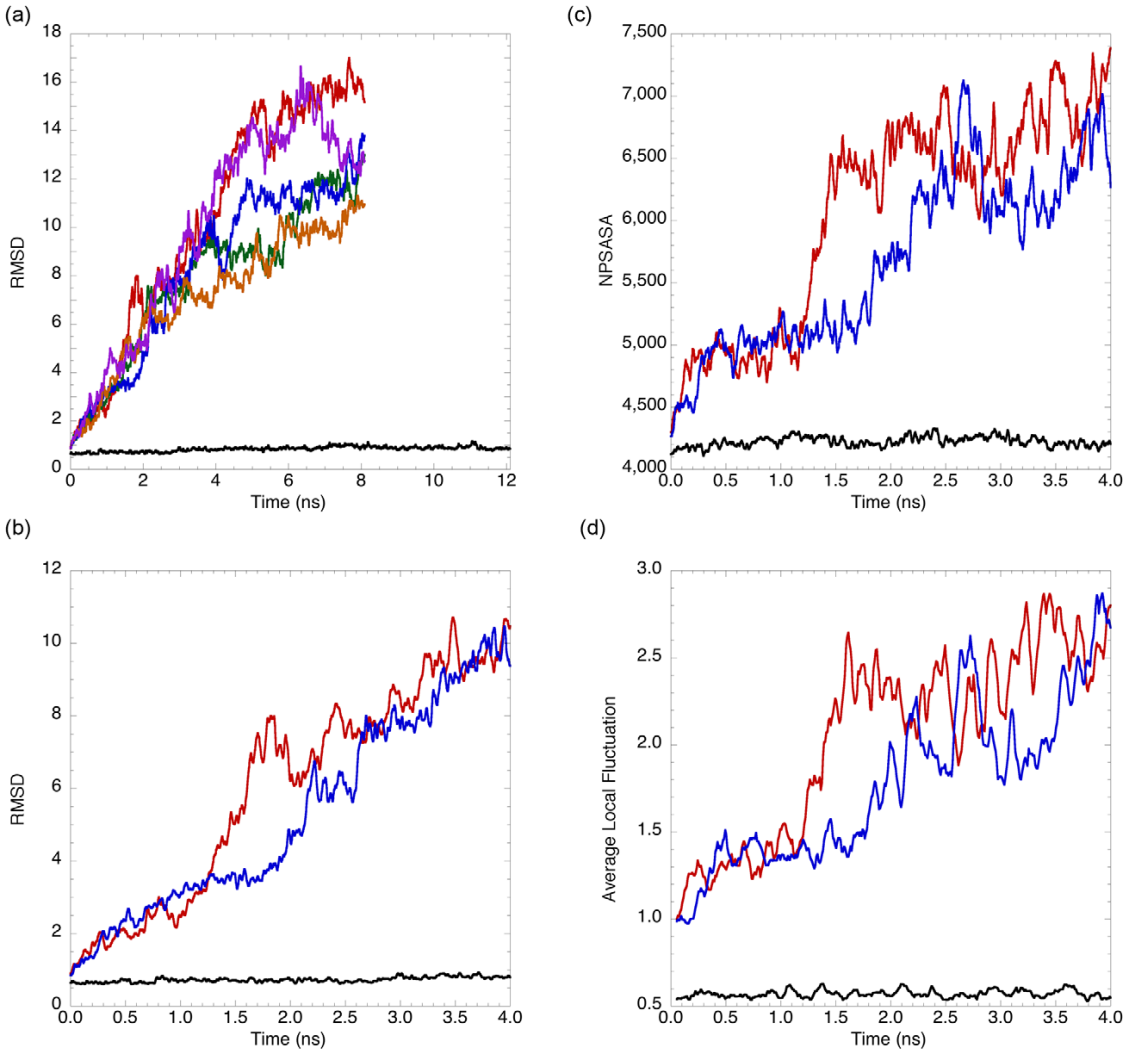
αLP unfolds significantly and reproducibly at high temperature but is stable at 298K. (a) At 500K, αLP unfolds quickly and fully in the five 8.1 ns unfolding simulations while it remains native-like at 298K as measured by Cα RMSD (black, 298K; red, 500K1; green, 500K2; blue, 500K3; orange, 500K4; purple, 500K5). (b,c,d) Colors used are the same as in (a). 500K1 and 500K3 were chosen due to the relatively large difference in their unfolding times. (b) Cα RMSD for the first 4 ns of 298K, 500K1, and 500K3 indicates unfolding occurs early at high temperature. (c) The NPSASA for the first 4 ns of 500K1, 500K3, and 298K is shown. After a short thermal equilibration, both 500K1 and 500K3 reach values ∼5000 Å^2^ and level off until exposing much more non-polar surface at 1.3 and 1.8 ns, respectively. At 298K, very little increase is seen in NPSASA. (d) ALF measures short-term fluctuations in structure and is an indicator of conformational flexibility of the molecule's current state. For both 500K1 and 500K3, conformational flexibility is low and then suddenly rises concurrently with NPSASA. ALF is low and stable at 298K. For all but (d), the data is smoothed with a 0.019 ns running average.

Simulations were performed with NAMD[24] using the CHARMM22[25] forcefield and TIP3P explicit water (full details in Methods). To test for proper behavior in our simulations, a 298K MD simulation of αLP was performed for 12.1 ns. αLP was quite stable, averaging 0.84 Å Cα RMSD to the crystal structure[26] over the course of the simulation and 0.87 Å Cα RMSD over the last 1 ns, with a maximum of 1.32 Å (Figure 2A). A previous 1 ns MD simulation of αLP at 300K using a different force field and simulation conditions also found little deviation from the crystal structure (average 0.83 Å Cα RMSD).[27] A long loop comprising residues 218–225 (Figure 1, middle right, orange) and several residues at turns contribute most of the differences and have higher than average B-factors in the crystal structure.[26] At 298K, there is little additional exposure of non-polar solvent accessible surface area (NPSASA), with an average increase of 5.5% in exposure (Figure 2C). It should be noted that the rigidity of αLP as seen by 298K simulation is considerably greater than what is observed for other proteins,[14],[18],[19] consistent with the very low crystallographic B-factors[26] and high hydrogen exchange protection factors[2] seen previously.

Five independent 8.1 ns MD simulations at 500K were conducted to determine the unfolding pathway of αLP, with the Cα RMSD of each plotted in Figure 2A. Visual inspection of the trajectories and the high Cα RMSDs attained indicated that αLP had unfolded in each simulation. By contrast, simulations at 450K showed little unfolding at similar timescales making them impractical for analysis (data not shown). Each trajectory shows a generally increasing Cα RMSD throughout the simulation, though there is significant variation in the rates of increase, periods of no change or decrease in Cα RMSD, and final Cα RMSD, as expected for independent simulations. Because relatively high RMSDs were reached in the first 4 ns of the simulations, we hypothesized that the major unfolding transition occurred in that timeframe (Figure 2B).

To confirm that unfolding had occurred, we examined molecular properties orthogonal to Cα RMSD early in the simulations. These properties, non-polar solvent accessible surface area (NPSASA) and a new metric termed Average Local Fluctuation (ALF), can distinguish native from non-native conformations without directly comparing them to the crystal structure. First, non-polar amino acid side-chains, normally buried in a protein's interior, become exposed upon unfolding, increasing NPSASA. The NPSASA for the first 4 ns of 298K1 (for comparison), 500K1, and 500K3 is plotted in Figure 2C. 500K1 and 500K3 were chosen for clarity due to a large difference in unfolding time. Both exhibit relatively small increases to ∼5000 Å^2^ within the first 0.3 ns, consistent with thermal equilibration. NPSASA then increases very slowly, unlike Cα RMSD, until it rapidly increases at 1.3 and 1.8 ns for 500K1 and 500K3, respectively. These sharp rises are followed by another slowly increasing phase that is highly variable for the rest of the simulations.

The second property, ALF, relies on the notion, derived from funnel energy landscape models of protein folding/unfolding, that molecules in the unfolded ensemble can explore many more conformations than those in the native ensemble.[28] For αLP, where the unfolding barrier has been shown experimentally to be extremely high, cooperative, and entropic in nature, it is certain that conformational space on the folded side of the TSE is quite restricted relative to the unfolded side.[2],[5] If unfolding simulations capture this ensemble behavior, there would be bottlenecks or barriers in the unfolding landscape. ALF was created to assay for these barriers, as it measures the rate of conformational change throughout a simulation (details in Methods). ALF for the first 4 ns of 298K1 (for comparison), 500K1, and 500K3 is plotted in Figure 2D. In the first 0.3 ns of both simulations, ALF increases slightly from 1.0 to 1.3 Å due to thermal equilibration. It remains relatively flat until rapid increases beginning at 1.3 and 1.8 ns for 500K1 and 500K3, respectively, resulting in a permanently higher ALF. In 500K3, ALF increases less sharply relative to 500K1, rapidly decreasing and then recovering in the middle of its rise ∼2.0 ns, which has implications for identifying its TSE (see below). The large and permanent increases in conformational flexibility measured by ALF and their coincidence with similar increases in NPSASA are indicative of seeing true unfolding transitions.

**Figure 3:**
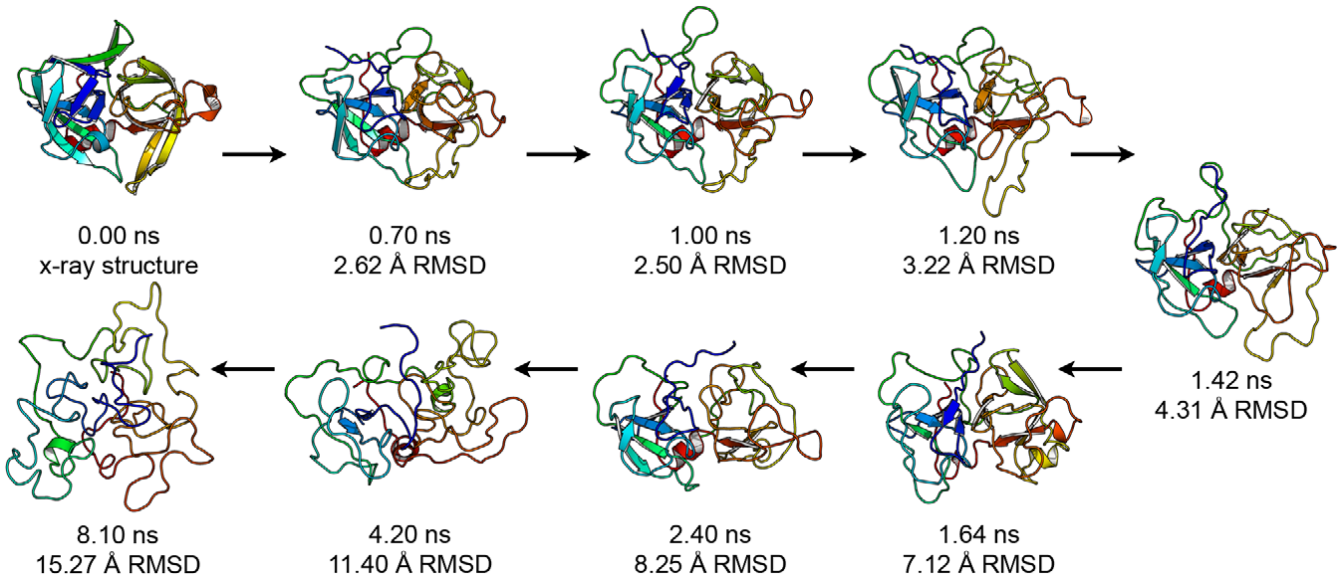
Selected structures from the 500K1 simulation illustrate the αLP unfolding pathway. Time in the simulation and Cα RMSD to the crystal structure are indicated.

Structurally, the early stages of αLP's unfolding pathway are quite consistent among the five unfolding simulations, though the simulations tend to diverge once the molecule becomes much less native-like. As we will show below, these early events constitute the major unfolding transition and are the primary focus of this work. First, we will describe the pathway in detail for 500K1, with several important conformations shown in Figure 3, and then note any important differences in other simulations. A movie of the full 500K1 unfolding pathway is shown in Video S1. For the first several hundred picoseconds, αLP thermally equilibrates and reaches ∼2 Å Cα RMSD to the crystal structure, with small surface loops the major source of this small deviation. At 0.7 ns, a large loop comprising residues 218–225 unique to αLP becomes more mobile, though its flexibility is somewhat limited by a disulfide bond between residues C189 and C220A. All residue numbering is based on homology to chymotrypsin, as in the PDB files. Because this loop is not conserved in kinetically stable proteases and is relatively mobile at 298K, we feel its overall impact on the unfolding pathway is small. At 1.0 ns, the Domain Bridge, a β-hairpin connecting the two domains of αLP, becomes more mobile but remains intact (Figures 1 and 3). Between 1.2 and 1.4 ns, αLP begins to unfold much more significantly, though the distortions are confined to four main structural areas: the N-terminal strand β1, the Domain Bridge, a region near the active site comprising the CβH and a *cis*-proline-containing turn (residues 91–102, CPT), and the 218–225 loop (Figures 1 and 3). β1 pulls away from the body of the protein and becomes highly flexible. The Domain Bridge breaks tertiary contacts with nearby residues and its two strands separate. Contacts between the CPT and the CβH break as the two pull away from each other, and the CβH strands separate. The 218–225 loop remains highly flexible, causing residues 215–217, which form part of the substrate binding groove, to separate from the β-barrel and push the CβH away from the body of the protein. These regions continue to unfold, accelerating the unfolding of nearby structure, though several regions remain relatively well-structured at 1.64 ns, including the β-sheets β4-β7-β6 and β14-β15-β16, and the C-terminal α-helix (Figure 3). The C-terminal β-barrel unfolds and further weakens the domain interface, with very few native-like interactions bridging the two domains at 2.4 ns (Figure 3). By 4.2 ns, little residual structure remains, as the Cα RMSD is 11.4 Å, though the molecule does continue to unfold, reaching a Cα RMSD over 16 Å within 8 ns (Figure 3). The presence of three disulfide bonds most likely prevents more extreme unfolding.

Early on, each of the unfolding simulations follows a similar trajectory to that of 500K1 although with variability in the timing (Figure 2), beyond this, some other differences do exist. In 500K4, β5 unfolds much earlier relative to the other simulations, separating from β2 and β6 and partially exposing the interior of the N-terminal domain to solvent. The turn connecting β5 to the more stable β6 (Figure 1, upper left, light blue) is quite flexible in all five unfolding simulations and has some of the highest B-factors in the crystal structure, which may explain part of this behavior.[7],[26] In 500K3, the Domain Bridge does break some tertiary contacts with surrounding regions early in unfolding, but its two strands separate relatively late. The N-terminal β1 does not completely separate from the body of the protein in 500K2 and 500K3 early on, as it does in the other three simulations, but its contacts are somewhat disrupted in both. Other differences at early time points appear to be relatively minor and are to be expected given five independent high temperature unfolding simulations.

### Determining the Location of the TSE

Because computational studies of protein unfolding are severely restricted in the number of molecules that can be simulated, they must use the vast amount of information present in each simulation in order to identify the TSE. As in other types of single-molecule experiments, there will be significant variation within the properties of the ensembles, such as time to unfold. Unlike experimental studies, where there is often a single reporter of the molecule's conformation, such as tryptophan fluorescence, MD simulations provide every conformation sampled, an enormous amount of data. However, there is no *a priori* way to say whether a particular three-dimensional structure is “folded” or “unfolded.” The challenge then is to derive properties from the conformations, either those directly computable from each structure or those that rely on comparing structures to each other, that can be used to clearly separate the folded from the unfolded conformations.

**Figure 4:**
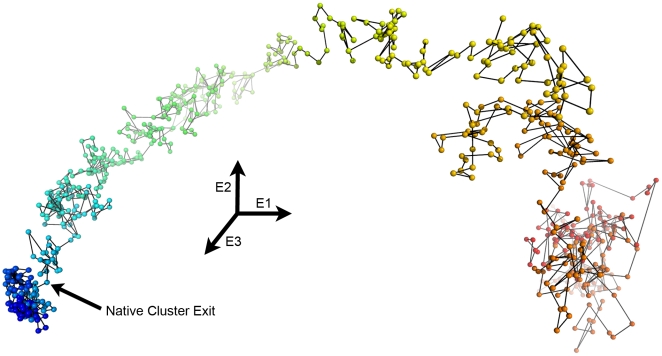
Conformational clustering effectively defines the exit from the native state. 3-D representation of conformational clustering of 500K1 generated by multi-dimensional scaling of the all-versus-all Cα RMSD. Each sphere is a conformation from every 10 ps of 500K1 and is connected by sticks to the preceding and following conformation. The earliest conformations are colored blue and the latest, red. E1, E2, and E3 represent the first through third eigenvectors from the multi-dimensional scaling. The exit from the native cluster is identified by the arrow and is at 1.41 ns.

Previous studies investigating the nature of a protein's TSE by unfolding simulations have often determined TSEs from individual simulations and combined them into an overall TSE.[15],[18],[29] These approaches depend on the assumption that the TSE is a small region of conformational space at the edge of the native basin, hence identifying them requires methods that clearly separate native from non-native conformations. One method that has had considerable success is a conformational clustering procedure pioneered by Li and Daggett.[13],[14] A pairwise Cα RMSD matrix is generated for all trajectory conformations and then projected down into two or three dimensions using multi-dimensional scaling. Visual clustering then separates the native conformations from the non-native, placing the TSE at the exit of the native cluster. While the method does require a significant level of subjective judgment, the Daggett group has had good success correlating results of their unfolding simulations to protein engineering studies of the same proteins. Conformational clustering was performed for each of the unfolding simulations here, with the three-dimensional projection of the 500K1 trajectory shown in Figure 4. Individual conformations extracted every 10 ps are shown as spheres and are connected chronologically by sticks; the color goes from blue to red as the simulation progresses. The first 1.41 ns of 500K1 is tightly clustered around the native state (lower left) and then rapidly moves away from the native state, forming much less dense clusters as it progresses through the simulation. Similar behavior is seen for the other unfolding simulations, allowing them to be effectively clustered (Table 1). However, it is much more difficult to identify a common TSE by conformationally clustering all five unfolding simulations simultaneously; hence we sought a method that would allow a common TSE to be generated, testing the conformationally clustered TSE.

**Table 1:** Time (ns) at the native cluster exit for the five αLP unfolding simulations.

Simulation	Conformational Clustering	NPSASA-Native Contacts	PCA Landscape
500K1	1.41	1.41	1.41
500K2	1.83	1.80	1.79
500K3	1.92	2.18	2.17
500K4	1.40	1.46	1.48
500K5	1.98	1.94	1.97

The only significant difference between the conformational clustering and the landscape methods is for 500K3.

**Figure 5:**
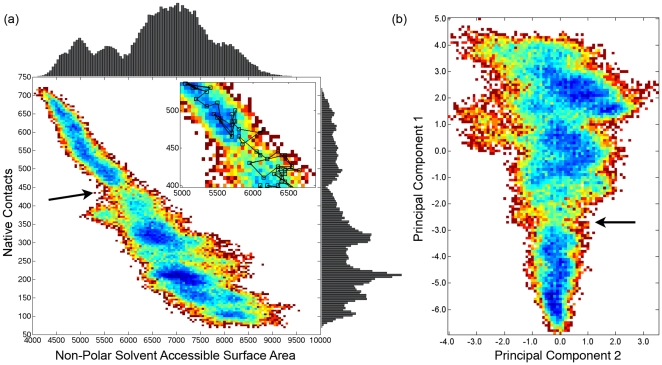
Property-based landscapes clearly separate native from non-native conformations. (a) The unfolding landscape is generated from all five unfolding simulations using native contacts and NPSASA as order parameters. 1-D histograms of native contacts and NPSASA are found to the right and above the landscape, respectively. The landscape was generated by taking the negative natural logarithm of the 2-D histogram with white being unobserved in the simulations, dark red the least populated, and progressing to dark blue as the most populated. The native state is in the upper left corner. A less populated region (indicated by the arrow) centered around 450 native contacts and 5900 Å^2^ separates native-like conformations from non-native conformations and represents the TSE. (inset) Zoomed-in view of TSE region, with trace of 500K1 overlaid. 500K1 crosses the TSE barrier only once and in less than 10 ps, between 1.41 and 1.42 ns; other simulations exhibit similar behavior. (b) Principal components analysis was used to reduced ten conformational properties to two dimensions (see Methods for list of properties). Coloring is the same as in (a). The native state is the well-populated region at the bottom of the figure and is separated from the non-native state by a barrier near −2.7 in PC1 (indicated by the arrow). Note that significant spread in PC2 is only seen after the TSE, as many more conformations are accessible in the unfolded state.

Although the ALF metric captures some of the significant changes during unfolding, it should be possible to gain a better picture of the unfolding process across all of the unfolding simulations, by not looking as a function of time, but rather through changing properties. Many structural properties, such as secondary structure content and fraction of native contacts, have been used to cluster trajectories or create energy landscapes both in unfolding simulations and in equilibrium simulations utilizing umbrella sampling.[15],[19],[22],[23],[30],[31] Using common protein folding/unfolding metrics (here, the number of native contacts and NPSASA) as order parameters, we have computed a single two-dimensional unfolding landscape that integrates data from all the simulations despite their individual differences in timing (Figure 5A). Histograms of the individual metrics are shown at the top and right of the landscape. The landscape shows three well-populated basins (dark blue), one native-like (upper left) and two progressively less native (middle and lower right). There is a bottleneck in the landscape, shown enlarged in the inset and centered around 450 native contacts and 5900 Å^2^ NPSASA, that separates the native from non-native basins. Also shown in the inset is a trace of the 500K1 simulation, at 10 ps intervals, for clarity (the landscape was constructed using conformations at 1 ps intervals, a total of 40500 conformations). Significantly, all simulations cross this bottleneck only once, implying a shared barrier to unfolding with these order parameters. The actual crossing transition occurs at different times in the different simulations, for example occurring between 1.41 and 1.42 ns for 500K1 (Table 1). We propose that this barrier is the location of the αLP TSE in these simulations and have generated a TSE from the structures making up the barrier (Table 1).

In reality, the αLP unfolding landscape is highly multi-dimensional and is only approximated by NPSASA and native contacts, which are clearly highly correlated. In order to utilize more of those dimensions, ten parameters were measured for each conformation (details and full listing in Methods). Principal components analysis (PCA) was used to eliminate the inherent correlations in the parameters and allow visualization in less than ten dimensions. The first two principal components explain 90% of the variance in the parameters and were used to generate a landscape as above (Figure 5B). Again, the region comprising native-like conformations is well-separated from the non-native region by a sparsely populated barrier centered around −2.7 on PC1 and 0.0 on PC2. Crossing times for all of the simulations are within 30 ps of the crossing times in the NPSASA/native contacts landscape, and, as above, we have generated a TSE from the PCA landscapes (Table 1). The first principal component, which contains relatively equal weightings from all ten parameters, is mostly a function of each conformation's nativeness (Table S1). There is little variation in the second principal component in the native-like region, and the simulation trajectories begin to diverge more significantly upon reaching the unfolding barrier. The second principal component is dominated by the size of the molecule and backbone exposure to solvent, as the three largest components are non-native mainchain hydrogen bonds, polar SASA, and radius of gyration (Table S1).

It is important to note that the landscapes in Figure 5 are not free energy landscapes[23], as the simulations analyzed here are non-equilibrium simulations, but represent the degree of sampling of the relevant structural properties. While interpretation of these landscapes is not as straightforward as that for free energy landscapes, we believe that they accurately identify the TSE. Unfolding should proceed rapidly once the TSE is passed in an individual simulation, as seen by the ALF metric (Figure 2D), which will limit sampling of the TSE region. Here, we have performed five independent simulations, observing a shared region in parameter space that is under-sampled and on pathway to the unfolded state. Importantly, the simulations only cross this region once, as expected given the strongly unfolding conditions. This coincidence of under-sampled parameter space for the combination of five simulations almost exactly coincides with the native exit cluster based on pair-wise structural comparisons for four of the five simulations, with a small error for 500K3. Agreement between such quite different methods is not a given, as has been observed in simulations of spectrin R17.[19] It is likely that the remarkable agreement seen here between conformational clustering and the landscape methods is due to the high cooperativity of αLP unfolding, which is experimentally observed[2]. Finally, we believe the PCA-landscape-derived TSE is the most accurate one, as its clustering is the least subjective, which may be an issue with 500K3 conformational clustering, the crossings of its observed barriers are unambiguous, and its generation from all five simulations adds additional evidence to its relevance.

### Unfolding Pathway and the TSE

**Figure 6:**
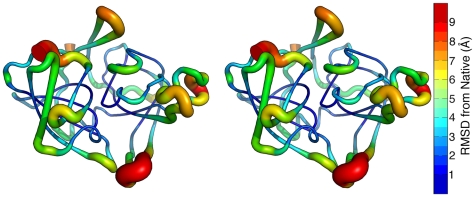
The structure of the αLP TSE. Deviations from native in the αLP TSE are restricted to several regions, mostly in the domain interface. Stereo view of the average Cα RMSD at each residue in the PCA landscape TSE from the crystal structure mapped onto the crystal structure. Both the thickness of the cartoon and the color indicate the deviation from native, with thicker representations meaning larger deviations.

For the remainder of this work, the αLP TSE is derived from the PCA landscape, generated by taking the conformations spanning the barrier crossing for each of the individual simulations (10 ps, conformations saved at 1 ps intervals) and combining them, yielding a TSE with 50 conformations. Some general properties of the TSE are listed in Table S2. Due to heterogeneity in large portions of the molecule, it is difficult to visualize the entire set of conformations (representative members are shown in Figure S1). As one way of visualizing the TSE, all TSE conformations and the crystal structure were superimposed using the structural superposition program THESEUS and the average deviation from the crystal structure at each Cα over all conformations was computed.[32],[33] These deviations were then mapped onto the crystal structure by color and thickness of the tube used to represent the backbone, as seen in Figure 6. Several observations can be made from this representation. First, significant deviations from the crystal structure are confined to several regions, notably those mentioned above. Much of the molecule is quite native-like, including the sheet β2-β3-β4-β7-β6 in the N-terminal domain and most of the β-barrel in the C-terminal domain. Second, as evident in stereo, the “front” face of αLP as depicted deviates far more from native than the “back” face. The “front” face contains the active site and these deviations would severely disrupt enzymatic activity. In addition, preliminary native state hydrogen exchange experiments found that denaturing agents had a more significant effect on the “front” face of αLP.[34] Third, with the exception of the 218–225 loop, which is not conserved, unfolding of the regions identified in each of the unfolding simulations, β1, the Domain Bridge, and the active site hairpins, would disrupt the domain interface and expose much of it to solvent.

### The Domain Interface's Role in Unfolding

**Figure 7:**
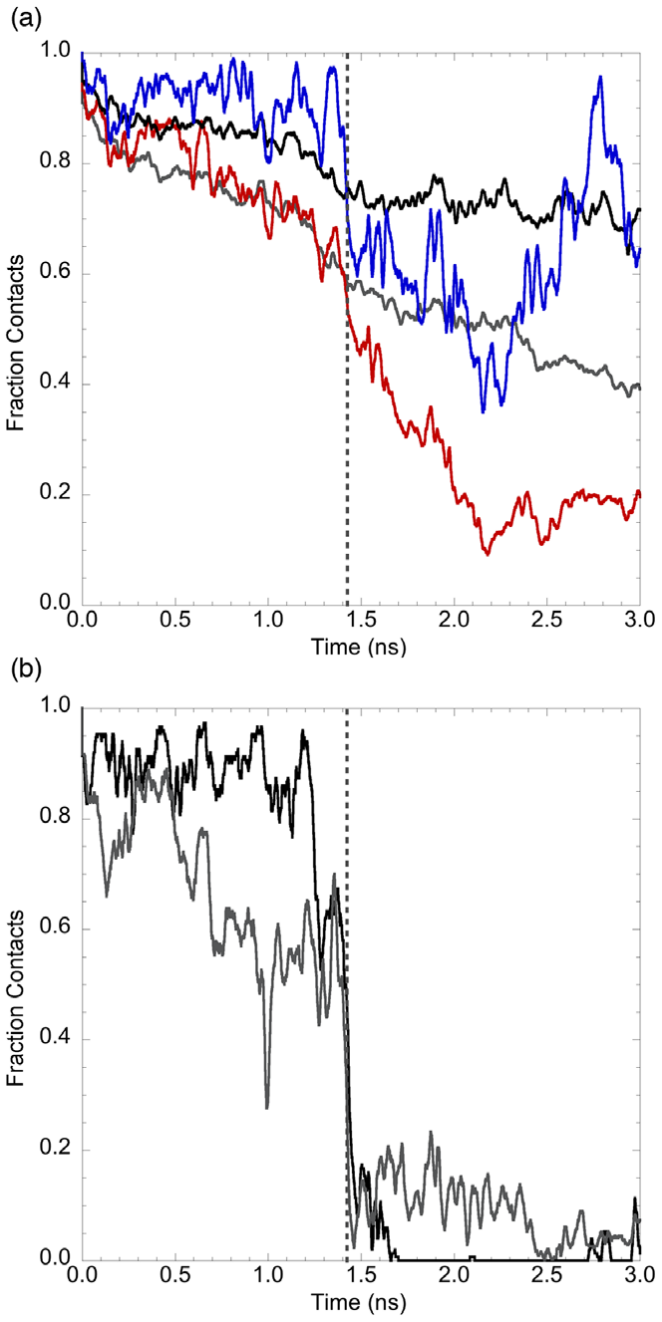
Contacts at the domain interface are preferentially broken at the unfolding transition. (a) The fraction of intra-domain (black), inter-domain (blue), native intra-domain (gray), and native inter-domain (red) are shown for the first 3 ns of 500K1. Inter-domain contacts experience a sharp drop at the native cluster exit (dashed vertical line, 1.41 ns) and continue to decline. Intra-domain contacts are lost more gradually. Shortly after unfolding, ∼90% of native inter-domain contacts are lost permanently. (b) The fraction of native domain bridge-domain bridge (black) and native domain bridge-other (gray) contacts for the first 3 ns of 500K1. Both decline sharply at the native cluster exit (dashed vertical line) and do not return to native-like values. For both (a) and (b), the data is smoothed with a 0.019 ns running average.

The entropic nature of the αLP unfolding barrier previously led us to hypothesize a solvated domain interface at the TSE, and investigations of the pH-dependence of unfolding has lent credence to that model.[5],[6] The TSE model presented here (Figure 6) is consistent with the individual domains remaining well-folded throughout the unfolding transition, but is seemingly at odds with the hypothesis that the domains open up in the TSE. To better investigate the domain interface's response to unfolding, we calculated the number of residue-residue intra-domain and inter-domain contacts present in each simulated conformation and normalized them by the corresponding number present in the crystal structure (shown for 500K1 in Figure 7A). Note that at the TSE (1.4 ns), the drop in inter-domain contacts is much more steep and continues much longer than the relatively shallow drop in intra-domain contacts. This effect is exaggerated if only contacts present in the crystal structure are considered. Gray and red curves represent these native intra-domain and inter-domain contacts, respectively. As before, native inter-domain contacts are being lost much more quickly at the TSE. At 2.0 ns, just 0.6 ns after the TSE, only 15% of native inter-domain contacts remain while 50% of native intra-domain contacts are present. This general pattern holds for the other unfolding simulations, providing additional evidence that a key step in αLP unfolding is the opening of the domain interface.

The Domain Bridge is an integral part of the αLP domain interface and has been experimentally implicated as a determinant of the unfolding rate.[7] To quantify its role in unfolding, we have calculated the normalized number of native contacts it makes, as above, though using atom-atom contacts due to the relatively small number of residues. Plotted in Figure 7B is are the fraction of native contacts between two residues both in the Domain Bridge (DB-DB, black) and between one residue in the Domain Bridge and any other residue (DB-O, green) for the first 3 ns of 500K1. DB-DB contacts are quite stable until the molecules begins to unfold significantly at 1.2 ns, reaching about 60% of native, and then losing all native contacts right at the TSE at 1.41 ns. DB-O contacts are lost more gradually prior to the TSE than DB-DB contacts, but they experience the same steep loss at the TSE. With the exception of 500K3 as noted above, the other unfolding simulations exhibit similar behavior. The high unfolding cooperativity of the Domain Bridge and its coincidence with the TSE observed here is consistent with the previous experimental studies.

### Unfolding Cooperativity

A critical feature for αLP's kinetic stability is its extremely high unfolding cooperativity. Previous work has shown that while αLP and trypsin, a thermodynamically stable homolog, have similar unfolding rates, αLP unfolding is much more cooperative as measured by proteolysis, providing it a functional advantage in highly proteolytic environments.[2],[3] Because determining the origins of this remarkable difference is crucial for understanding the molecular basis for kinetic stability, we sought to compare the behaviors of αLP and trypsin as revealed by unfolding simulations. Four 10.1 ns unfolding simulations at 500K were performed for trypsin. Although a thorough discussion of the details of the trypsin TSE and unfolding pathway will be presented elsewhere, the general behavior of these simulations is reported in the Supplementary material (Figures S2 and S3, Table S3).

**Figure 8:**
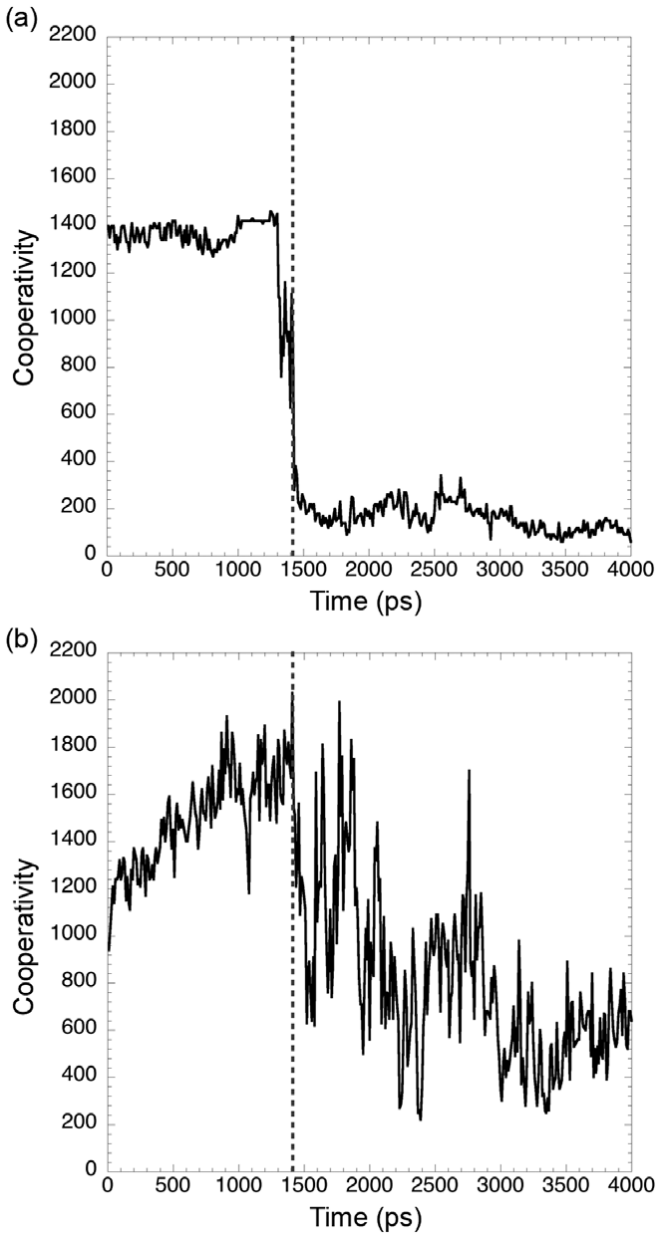
αLP unfolds significantly more cooperatively than trypsin. Cooperativity is measured by counting the number of sampled conformations <3 Å Cα RMSD (two-fit Cα RMSD, see Methods) from the conformation at each time point. (a) Cooperativity for the first 4 ns of 500K1. Starting flat and steeply dropping indicates a very cooperative unfolding transition for αLP. (b) Cooperativity for the first 4 ns of 500K2T (trypsin). Trypsin unfolds much less cooperatively than αLP, as seen by the gradual rise early in the simulation and the gradual and noisy decline starting at 1.4 ns. (a) and (b) Vertical dashed line indicates position of the native cluster exit in each simulation.

To quantitatively compare unfolding cooperativity, we developed a new metric defined by how many conformations were similar (based on a Cα RMSD threshold) to the *i*th conformation within the *n* total simulation conformations. The cooperativity graph for a perfectly cooperative unfolding transition would be high and flat for the beginning of the simulation, drop steeply at the TSE, and then be much lower for the duration of the simulation. Specifically, it would have a value of *j* from 1 to *j*, where *j* is the TSE conformation, and drop to a value *k*≪*j* after the TSE. Less cooperative transitions would feature gradually increasing and/or decreasing values prior to the TSE and less steep drops after the TSE. Cooperativity for αLP (500K1) and trypsin (500K2T) are shown in Figure 8A and 8B, respectively. The cooperativity profile for αLP is very similar to that of the hypothetical perfectly cooperative unfolding transition. Before the TSE at 1410 ps, the value is near 1400 and relatively flat. It drops sharply right at the TSE, and then is much lower for the duration of the simulation. Trypsin, on the other hand, unfolds much less cooperatively. Its increasing profile from 0 to 900 ps represents gradual unfolding, because structures that have partially unfolded are similar to both the native structure and to more unfolded conformations. It has no clear steep drop from the native state as αLP does, only a gradual and very noisy decline. Its values post-TSE are much higher than those observed for αLP, which suggests a more rugged, gradual unfolding process. Cooperativity plots for the other simulations show the same general trends and the behavior is qualitatively similar with different choices of Cα RMSD thresholds. We believe this work is the first example of both measuring cooperativity in simulated unfolding and comparing it across two proteins where that difference has functional relevance.

## Discussion

A major motivation for this study was providing atomic resolution to previous biochemical experiments on αLP unfolding, but first those lower resolution results must be reproduced. A comprehensive analysis of experimental data on protein unfolding barriers revealed a stark difference between those of αLP and thermodynamically stable proteins: the αLP unfolding barrier is significantly more entropic, suggesting the αLP TSE is considerably more native-like than those for thermodynamically stable proteins.[5] In addition, m-value analysis of unfolding found the fraction of SASA buried at the αLP TSE was computed to be 80%, also highly-native like.[35] The simulation-derived TSE reported here is quite similar to the native structure, with an average Cα RMSD of 4.4±0.4 Å and with 38% of Cα atoms being less than 2.0 Å from native. The average fractional SASA at the TSE is 82±2%, slightly higher but quite consistent with the value derived from experiment. One possibility for the slight deviation is that the elevated temperature in the simulations shifts the TSE somewhat towards the native, a modest Hammond effect that was seen for CI2.[36],[37]

One of the challenges in this study is to identify a robust TSE from the unfolding trajectories at high temperatures. The most widely accepted definition of the TSE is the p_fold_ definition, which defines the TSE as the ensemble of states in which at physiological temperatures, half the states refold and the other half unfolds. In recent studies of two very small domains, CI2 [38] and the engrailed homeodomain [39], it was demonstrated that conformations from the TSE identified by multi-dimensional scaling in high temperature unfolding simulations satisfies the p_fold_ definition at physiological temperatures. Indeed, at temperatures near the transition temperature, these conformations are seen to oscillate between the folded and unfolded states [40]. However the p_fold_ verification required 36000 times longer simulation time than the original unfolding simulation, a calculation that would not be feasible for αLP, a much larger protein. Once practical, it would be useful to carry out such calculations. Nevertheless, based on the studies above, we believe that the TSE of αLP identified here represents a useful estimate of the physiological TSE. One reason is that in the PCA landscape of αLP (Figure 5), there is a particularly tight bottleneck that separates the folded and unfolded states. As significant unfolding trajectories must pass through this bottleneck, the TSE must be found near the bottleneck. Extrapolating this behavior to physiological temperatures, we expect this bottleneck to become even more pronounced, thereby localizing the TSE in conformation space. Moreover, there is strong connection between inferences developed from the calculated TSEs and a wide range of experimental observations with αLP, providing further confidence that the calculated TSEs provide relevant insight into the TSEs at physiological temperatures.

Previous experiments have shown a large role for the domain interface in αLP unfolding. The thermodynamic analysis referenced above suggested a possible model for the TSE: a “cracked egg” where the two β-barrel domains are largely intact but the extensive domain interface between them is disrupted.[5],[6] Relocation of salt bridges spanning the domain interface significantly decreased αLP's sensitivity to low pH unfolding, consistent with the “cracked egg” model,[6] (P. Erciyas, private communication). The simulations presented here confirm the disruption of the domain interface at the TSE, provide atomic detail as to how it happens, and extend these insights to two other critical structural regions: β1 and the CPT and CβH.

The Domain Bridge, the covalent linkage between αLP's two domains, has been shown to modulate the unfolding rate.[7] The simulations support this; they reveal that many of its native contacts are lost at the TSE, including separation of its strands, allowing it to make non-native contacts. The domain bridge makes several contacts with the N-terminal β-strand β1, which is also significantly disrupted at the TSE. Our results indicate a probable coupling of the unfolding of the Domain Bridge and β1, though the coupling is less evident in 500K2 and 500K3. When full-length Pro-αLP is synthesized, the C-terminus of the Pro region is covalently connected to the protease's N-terminus. As the protease domain folds, it gains proteolytic activity, cleaving the Pro-αLP junction that is positioned across the active site.[41],[42] The active site is 20 Å away from the location of the N-terminus in the native state and hence folding requires a significant rearrangement of the N-terminal strand. The flexibility of the N-terminus at the TSE in our simulations is consistent with its requirements during Pro-assisted folding. The last region at the domain interface disrupted at the TSE forms part of the active site.

Previous studies on αLP have also implicated the CβH as important to the folding/unfolding landscape. Mutations in the hairpin affected both the unfolding rate and the Pro-catalyzed folding rate.[8],[43] The Pro-αLP complex structure revealed that this hairpin forms a larger five-stranded β-sheet with Pro; mutants disrupting the interface there significantly weaken Pro's foldase activity.[42] The hairpin forms several side-chain contacts and two main-chain hydrogen bonds with the CPT in the native state; CPT residues F94, which forms the bulk of the contacts with the CβH, and *cis*-P95 are both completely conserved in kinetically stable proteases. The amides in these hydrogen bonds have relatively weak protection factors compared to the rest of the protein, consistent with them being broken at the TSE.[2] These contacts are also relatively long-range in sequence space, requiring that the molecule must give up significant conformational entropy in bringing them together, again arguing that they are broken early in unfolding. In our simulations, once the contacts between the two structures are broken, CβH pulls away from the body of the protein and its strands separate; the presence of the Pro region would keep the hairpin in a position ready to make contacts with the *cis*-proline turn, stabilizing the TSE. By understanding the αLP unfolding pathway and TSE in atomic detail, we can begin to explore how the Pro region stabilizes the TSE and accelerates folding 10^9^-fold.

The three regions of the domain interface disrupted at the TSE have something else in common: they are only found in the kinetically stable proteases and not the thermodynamically stable family members, such as trypsin. αLP and trypsin are good structural homologs; 120 (of 198) αLP's Cα's have an equivalent position in trypsin, with 99 of them within 2.0 Å of their trypsin equivalent.[44] It seems an unlikely coincidence that the regions of αLP that unfold at the TSE happen to be in the 1/3 of the protein that is not homologous to trypsin. In fact, for both the αLP and trypsin simulations, the structurally conserved regions are much more native-like at the TSE and beyond than are the non-conserved regions. Significantly, large parts of the αLP domain interface are made up of the non-conserved regions, likely resulting in the dramatic differences observed between folding of individual αLP and trypsin domains. For both chymotrypsin and trypsin, the two domains fold independently and upon mixing will form the active enzyme.[45],[46] By contrast, active αLP cannot be reconstituted from unfolded individual domains even in the presence of the Pro region.[47] αLP's cooperativity in folding echoes that of the unfolding reaction and likely involves the Domain Bridge and other regions of the domain interface which are distinct from its metazoan homologs.

**Figure 9:**
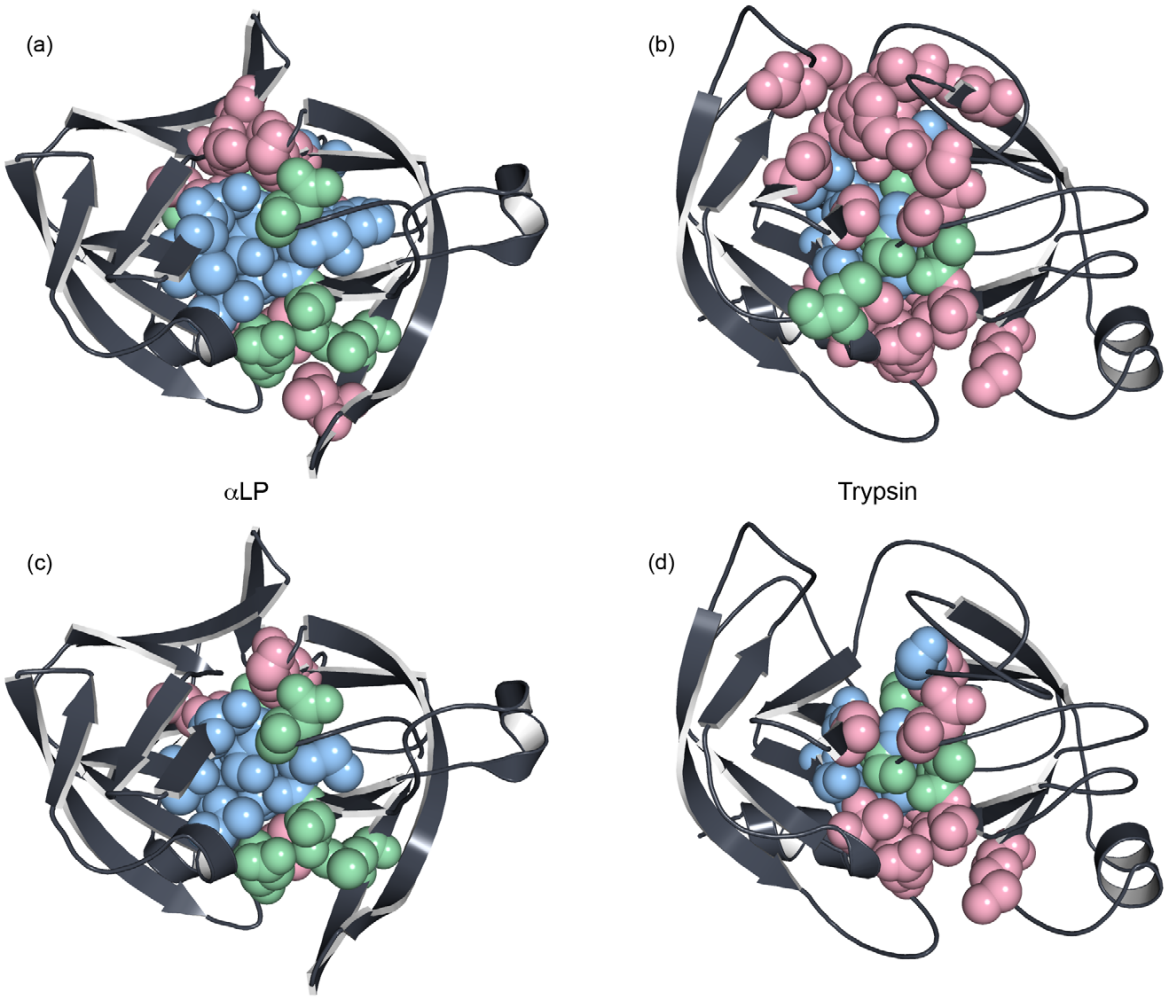
Solvation of the domain interface during unfolding differs significantly between αLP and trypsin. (a) - (d) Residues colored light red are solvent-exposed at the TSE, light green residues become exposed within 600 ps of the TSE, and light blue residues are still buried 600 ps past the TSE. (a) and (c) αLP, (b) and (d) trypsin. (a) and (b) All buried domain interface residues, (c) and (d) the subset of (a) and (b) where the position is conserved and found at the domain interfaces of both prokaryotic and metazoan proteases. Notably, many fewer buried residues of the αLP domain interface are solvated at the TSE compared to trypsin, even after eliminating the non-conserved positions.

By closely examining the differences between the domain interfaces of αLP and trypsin, we can begin to discover the mechanism of αLP's unfolding cooperativity. The buried residues (less than 5% exposed in the crystal structure) of the αLP and trypsin domain interfaces are shown in space-filling spheres in Figure 9A and 9B, respectively. Residues colored light red are exposed to solvent at the TSE, while residues still buried at the TSE are further subdivided into light green and light blue residues, which at 600 ps post-TSE are either exposed to solvent or still buried, respectively. For both αLP and trypsin, residues near the “top” and “bottom” of the molecules are more likely to be exposed at the TSE, while the “middle,” which contains the conserved active site, has fewer red residues. Clearly, more of trypsin's buried domain interface residues are exposed to solvent at the TSE than for αLP. In addition, the αLP core is much more blue than trypsin, as this core is much more resistant to solvation even post-unfolding than its metazoan counterpart.

As alluded to previously, much of the domain interface is not conserved between the two families of proteases. Figures 9C and 9D focus on the conserved interface, and correspond to 9A and 9B, respectively, after removing all residues that are not common to both domain interfaces (this implies position and sequence conservation). Here, the difference between αLP and trypsin is striking; over half of the positions in trypsin are red (solvent exposed), while residues in αLP are generally exposed to solvent much later, over half of them blue. An area near the active site (see Figure 1) comprising αLP residues D102, L180, and S214 (all green) and their trypsin equivalents D102, M180, and S214 (all light red) is particularly interesting. Again, this region is composed of the CβH unique to the kinetically stable proteases, and though it unfolds at the TSE, only once it completely unfolds does it begin to exposed the conserved core to solvent. This is not the case in trypsin, where the different architecture, composed of loops, allows relatively small unfolding events to expose the buried interface. Moreover, unlike αLP, these unfolding events in trypsin must be uncoupled from one another resulting in much greater structural variability in the TSE or equivalently, multiple, parallel approaches to the TSE. Thus one can think of trypsin as slowly diffusing over a broad unfolding barrier whereas αLP's behavior is much more concerted and the passage over its barrier is more tightly constrained.

Examining the differences between the full-domain interface and the conserved domain interface figures then highlights the non-conserved regions. At the αLP Domain Bridge, its unfolding exposes relatively little of the domain interface at the TSE, while the much larger equivalent area in trypsin is quite solvated. An important difference between the two proteases is that the αLP Domain Bridge is a compact, cooperative substructure, a simple β-hairpin. In trypsin, the domain interface is formed by two long and relatively floppy loops, which are inherently less cooperative than the domain bridge. Many of the non-conserved domain interface residues in αLP are also in secondary structure or tightly constrained turns near the Domain Bridge or active site (i.e. G18 and G19 connect β1 to β2 and interact with the Domain Bridge, V120B and V121 form the base of the Domain Bridge, V177 is in the CβH), while in trypsin these areas are formed with much less constrained loops. The differences seen here in the Domain Bridge and active site regions provide evidence that extreme unfolding cooperativity is generated by using highly cooperative substructures to protect the rest of the domain interface from solvent.

Intriguingly, increased protease resistance mediated through high inter-domain cooperativity has been observed in an unrelated system.[48] A screen of the *Escherichia coli* proteome for protease resistance found 40 proteins, one of which was the glycolytic enzyme phosphoglycerate kinase (PGK).[49] Young et al. found that while the *E. coli* and *Saccharomyces cerevisiae* enzymes had similar stabilities, the yeast PGK unfolded and was degraded much faster than the *E. coli* PGK.[48] The difference was attributed to the domain interface; the separated domains of yeast PGK fold independently and are quite stable, unlike the *E. coli* PGK domains, analogous to the difference between prokaryotic αLP and eukaryotic trypsin.

Recent work from Hills and Brooks on flavodoxin fold proteins using Gō models offers relevant insight into dynamics and cooperativity.[31],[50] First, they showed that contact density, the ratio of native contacts to the number of residues within a subset of the protein, was an accurate predictor of the nucleating subdomain in flavodoxin folds. Intriguingly, increased contact density in a specific region in Spo0F was consistent with local rigidification relative to other flavodoxin folds; this extra contact density induced higher topological frustration during Spo0F folding simulations. Using the native contacts definition in the Methods, αLP has a higher overall contact density than trypsin (3.89 and 3.64); this difference is magnified when considering the buried domain interface residues (Figure 9) (6.23 and 5.62 for all, 6.12 and 5.37 for the non-conserved residues). The insights from contact density are again consistent with the previous experimental and computational results presented here. αLP is more rigid than trypsin and unfolds more cooperatively, stemming from the non-conserved regions of its domain interface, but its folding landscape is extremely frustrated, resulting in folding kinetics on the order of millennia instead of seconds.

The costs of evolving extreme unfolding cooperativity are high; for αLP, the bacterium must synthesize a 166 residue protein to catalyze αLP folding after which it is immediately degraded. αLP's extremely slow folding is a consequence of the large energy gap between its unfolded/molten globule states and the TSE.[1] One likely contributor that has been previously noted is its high glycine content, as glycines in formed structures lose much conformational entropy relative to unstructured glycines.[51] These glycines, which make up 18% of kinetically stable proteases, are used to form tight turns and tight packing in areas where even an alanine would be sterically hindered.[1],[26] Like most proteins, the metazoan proteases have much lower glycine content (about 9%) and have many correspondingly longer loops than the prokaryotic proteases. These loops, like those in the domain interface of trypsin, are likely the reason for trypsin's lack of cooperative unfolding.

Finally, the idea that a protein's folding transition state is determined by its native structure, as shown through studies of Contact Order and folding rates, poses interesting questions for this class of proteases.[52],[53] While trypsin fits well in the Contact Order plot, αLP is an extreme outlier, perhaps not surprising given its remarkably slow folding.[5] The two proteins have the same fold and would be expected to have similar TSEs. Here, we have identified the TSEs for both, and remarkably, those TSEs both contain much of the conserved core of the fold. However, the regions where the two proteases differ are critical parts of the TSE structures. While the general structure of the TSE may be mostly determined by the native structure, the details, such as highly cooperative units making up the domain interface in αLP and not trypsin, can provide large functional advantages depending on the environment of the particular protein.

## Materials and Methods

### Simulations

1SSX and 5PTP PDBs were used for αLP and trypsin, respectively. All non-protein and hydrogen atoms were removed and hydrogens were added back with XPLOR.[54] For residues with multiple conformations, the “A” conformation was used. Protein molecules were placed in cubic boxes with a minimum of 12 Å distance to the edge and solvated with TIP3P explicit water and chloride counter-ions using Packmol,[55] where the approximate density was determined by the density of liquid water at the corresponding temperatures.[56] The number of atoms for 298K αLP, 500K αLP, 298K trypsin, and 500K trypsin were 32760, 28005, 33223, 28468, respectively. All simulations were performed using NAMD 2.5 with the CHARMM22 forcefield.[24],[25] Simulations were carried out with periodic boundary conditions, a 12 Å cutoff for non-bonded interactions, and Particle Mesh Ewald for long-range electrostatics. A timestep of 1 fs was used and snapshots were saved every 1 ps. Each system was equilibrated using the following protocol. The protein was fully constrained and the solvent was minimized for 500 steps using a conjugate gradient algorithm. The solvent was equilibrated for 100 ps under NPT conditions (298K and 1.01325 bar or 500K and 27 bar) using Berendsen coupling for both pressure (100 fs relaxation time) and temperature (2.0 ps coupling constant).[57] The solvent was then fully constrained and the protein was minimized for 50 steps. The entire system was then minimized for 50 steps. Finally, the system was equilibrated for 100 ps under the same NPT conditions. Multiple independent simulations were generated by starting the whole-system equilibration using different random number seeds for each. After equilibration, production simulations were carried out in the NVE ensemble, with the box size fixed at its final size from the equilibration. One 298K αLP (12.1 ns), five 500K αLP (8.1 ns each), one 298K trypsin (3.6 ns), and four 500K trypsin (10.1 ns each) simulations were performed for 96.6 ns total simulation time.

### Two-fit Cα RMSDs

In several analyses presented here (Conformational Clustering, ALF, and Cooperativity), Cα RMSDs were calculated with two fits to the target structure in order to lessen the impact of a small number of poorly aligning residues. Structures were aligned using all Cα atoms and the mean and standard deviation of the deviations were calculated. Cα atoms whose deviations were greater than two standard deviations above the mean were discarded for the second fit and calculation of Cα RMSD. This fitting procedure eliminated an average of 5% of the Cα atoms.

### Average Local Fluctuation (ALF)

For all overlapping 90 ps windows in a simulation, all pairwise two-fit Cα RMSDs were calculated for the 10 snapshots (10 ps intervals), resulting in 45 two-fit RMSDs at 801 windows for an 8.1 ns simulation. These RMSDs were averaged to give the ALF at the midpoint of each window. ALF therefore measures the extent of short-timescale (90 ps) fluctuations throughout the simulation, as it is the mean RMSD between any two snapshots within a short time window.

### Conformational Clustering

For each simulation, pairwise two-fit RMSDs were calculated at 10 ps intervals, forming a symmetric N × N matrix, with N = 810 for αLP and N = 1010 for trypsin unfolding simulations. Multi-dimensional scaling, as implemented in the MATLAB Statistical Toolbox,[58] was used to calculate the first three eigenvectors of the RMSD matrix. The resulting three-dimensional graph, where each point represents a single conformation, was visually clustered to identify the native state ensemble and its exit.

### Contacts

Atoms less than 4.6 Å apart or 5.4 Å apart if one of the atoms was C or S and more than two residues separated in the primary sequence were judged to be in contact. A contact was defined as native if the two residues had a contact in the crystal structure. For the purposes of defining inter-domain, intra-domain, and domain bridge contacts in αLP, the N-terminal domain is residues 15A-120E and 231–245, the Domain bridge is residues 120A-121, and the C-terminal domain is residues 120G-230.

### Native Contacts-NPSASA Landscape

For each simulation snapshot, the number of native residue-residue contacts and the NPSASA were calculated. The values were binned into a two-dimensional histogram using bin sizes of 5 native contacts and 50 Å^2^. The landscape was generated by taking the negative natural logarithm of the bin counts at each position.

### Principal Components Landscape

Ten conformational properties were used to generate the landscape: Cα RMSD, native intra-domain atom-atom contacts, native inter-domain atom-atom contacts, non-native intra-domain atom-atom contacts, non-native inter-domain atom-atom contacts, radius of gyration, non-polar SASA, polar SASA, non-native main-chain hydrogen bonds, native main-chain hydrogen bonds. Properties were scaled by dividing by subtracting the mean value and dividing by the standard deviation for each. Principal components analysis was performed with the MATLAB Statistics Toolbox. Loadings for each term in the PCA are shown in Supplemental Table 1. A two-dimensional histogram was computed using the first two principal components, with a bin size of 0.1 units. The landscape was generated by taking the negative natural logarithm of the bin counts at each position.

### Cooperativity

Two-fit Cα RMSDs were calculated for each pair of snapshots (10 ps intervals to reduce the number of pairwise comparisons) in a simulation. Cooperativity was defined as the number of snapshots less than 3 Å of the above Cα RMSD at each time point in the simulation multiplied by the snapshot interval (10 ps). Results were qualitatively similar using thresholds of 3.5 and 4.0 Å.

### Molecular Graphics

PyMOL[59] was used to generate Figures 1, 3, 4, 6, and 9.

## Supporting Information

Table S1 Parameter loadings for the αLP Principal Components Analysis landscape.
(0.04 MB DOC)

Table S2 Selected properties of the αLP crystal structure and TSE. Means ±1 standard deviation are shown for each TSE.
(0.04 MB DOC)

Table S3 Properties of the trypsin TSE. The trypsin TSE was generated using the conformational clustering method due to the heterogeneity of the unfolding simulations.
(0.03 MB DOC)

Figure S1 Representative conformations of the αLP TSE from each simulation show both the similarity and diversity of the TSE. The structures are colored blue at the N-terminus and progressing to red at the C-terminus.
(0.50 MB TIF)

Figure S2 Cα RMSD for trypsin control and unfolding simulations (black, T298K; red, T500K1; green, T500K2; blue, T500K3; orange, T500K4).
(0.26 MB TIF)

Figure S3 X-ray structure of trypsin and members of its unfolding TSE from each simulation. Some similarities are seen with αLP, particularly the maintenance of the β-sheet in the N-terminal domain and the C-terminal α-helix and the disruption of the domain interface both near the active site and at the “top” of the molecule as pictured.
(0.57 MB TIF)

Video S1 The entire 500K1 unfolding trajectory. The molecule is colored blue at the N-terminus and progressing to red at the C-terminus. Conformations every 2ps are shown. The TSE occurs near the 30 second point in this video.
(10.75 MB MOV)
