# Supplementary material for: Unfolding Simulations Reveal the Mechanism of Extreme Unfolding Cooperativity in the Kinetically Stable α-Lytic Protease
Source: PLoS Comput Biol. 2010 Feb 26;6(2):e1000689. doi: 10.1371/journal.pcbi.1000689 (PMC2829044; doi:10.1371/journal.pcbi.1000689)
Supplement: Table S1 — Parameter loadings for the αLP Principal Components Analysis landscape. (0.04 MB DOC) [file pcbi.1000689.s001.doc]

| Parameter | PC1 | PC2 |
| --- | --- | --- |
| Cα RMSD | 0.344 | -0.061 |
| Native Intra-Domain Contacts | -0.350 | 0.009 |
| Native Inter-Domain Contacts | -0.329 | -0.108 |
| Non-Native Intra-Domain Contacts | 0.323 | 0.280 |
| Non-Native Inter-Domain Contacts | 0.268 | 0.351 |
| Radius of Gyration | 0.312 | -0.364 |
| Non-Polar Solvent Accessible Surface Area | 0.332 | -0.267 |
| Polar Solvent Accessible Surface Area | 0.284 | -0.534 |
| Native Backbone Hydrogen Bonds | -0.346 | -0.052 |
| Non-Native Backbone Hydrogen Bonds | 0.259 | 0.539 |
