# Supplementary material for: Unfolding Simulations Reveal the Mechanism of Extreme Unfolding Cooperativity in the Kinetically Stable α-Lytic Protease
Source: PLoS Comput Biol. 2010 Feb 26;6(2):e1000689. doi: 10.1371/journal.pcbi.1000689 (PMC2829044; doi:10.1371/journal.pcbi.1000689)
Supplement: Table S2 — Selected properties of the αLP crystal structure and TSE. Means ±1 standard deviation are shown for each TSE. (0.04 MB DOC) [file pcbi.1000689.s002.doc]

| Simulation | Cα RMSD (Å) | NPSASA (Å^2^) | Native Contacts |
| --- | --- | --- | --- |
| Native | 0.00 | 4005 | 771 |
| 500K1 | 4.39 ± 0.15 | 6110 ± 110 | 458 ± 6 |
| 500K2 | 4.93 ± 0.06 | 5680 ± 110 | 451 ± 8 |
| 500K3 | 5.98 ± 0.12 | 5690 ± 170 | 462 ± 10 |
| 500K4 | 5.04 ± 0.11 | 6220 ± 130 | 442 ± 6 |
| 500K5 | 5.23 ± 0.12 | 5820 ± 160 | 445 ± 9 |
| ALL | 5.1 ± 0.5 | 5900 ± 300 | 451 ± 11 |
