# Supplementary material for: Unfolding Simulations Reveal the Mechanism of Extreme Unfolding Cooperativity in the Kinetically Stable α-Lytic Protease
Source: PLoS Comput Biol. 2010 Feb 26;6(2):e1000689. doi: 10.1371/journal.pcbi.1000689 (PMC2829044; doi:10.1371/journal.pcbi.1000689)
Supplement: Table S3 — Properties of the trypsin TSE. The trypsin TSE was generated using the conformational clustering method due to the heterogeneity of the unfolding simulations. (0.03 MB DOC) [file pcbi.1000689.s003.doc]

| Simulation | Time at native cluster exit (ns) | TSE Cα RMSD (Å) | TSE Fraction Native Contacts |
| --- | --- | --- | --- |
| T500K1 | 3.64 | 5.16 ± 0.12 | 0.515 ± 0.008 |
| T500K2 | 1.41 | 5.11 ± 0.10 | 0.553 ± 0.010 |
| T500K3 | 1.04 | 5.91 ± 0.04 | 0.560 ± 0.011 |
| T500K4 | 1.57 | 5.59 ± 0.09 | 0.457 ± 0.012 |
| ALL |  | 5.4 ± 0.3 | 0.52 ± 0.04 |
