# Supplementary figures and images for: Unfolding Simulations Reveal the Mechanism of Extreme Unfolding Cooperativity in the Kinetically Stable α-Lytic Protease
Source: PLoS Comput Biol. 2010 Feb 26;6(2):e1000689. doi: 10.1371/journal.pcbi.1000689 (PMC2829044; doi:10.1371/journal.pcbi.1000689)

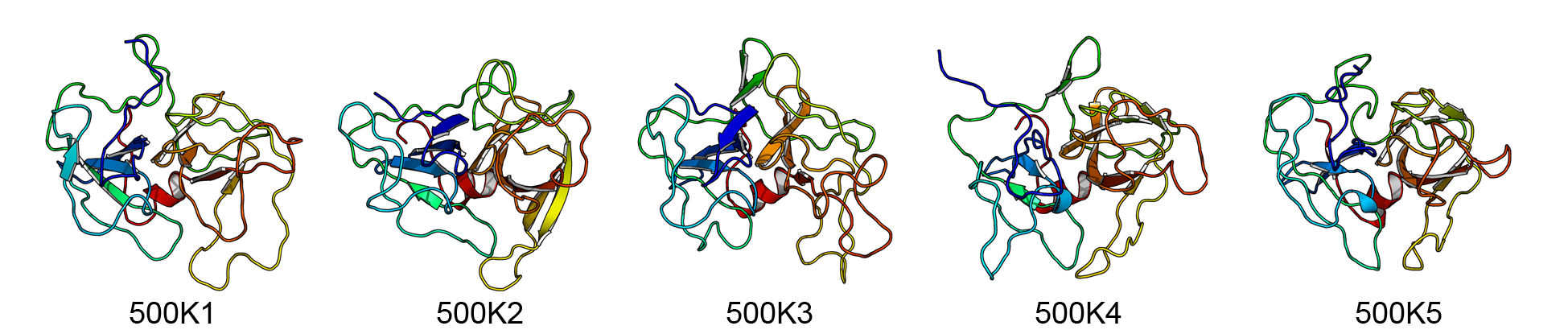

Supplement: Figure S1 — Representative conformations of the αLP TSE from each simulation show both the similarity and diversity of the TSE. The structures are colored blue at the N-terminus and progressing to red at the C-terminus. (0.50 MB TIF) [file pcbi.1000689.s004.tif]

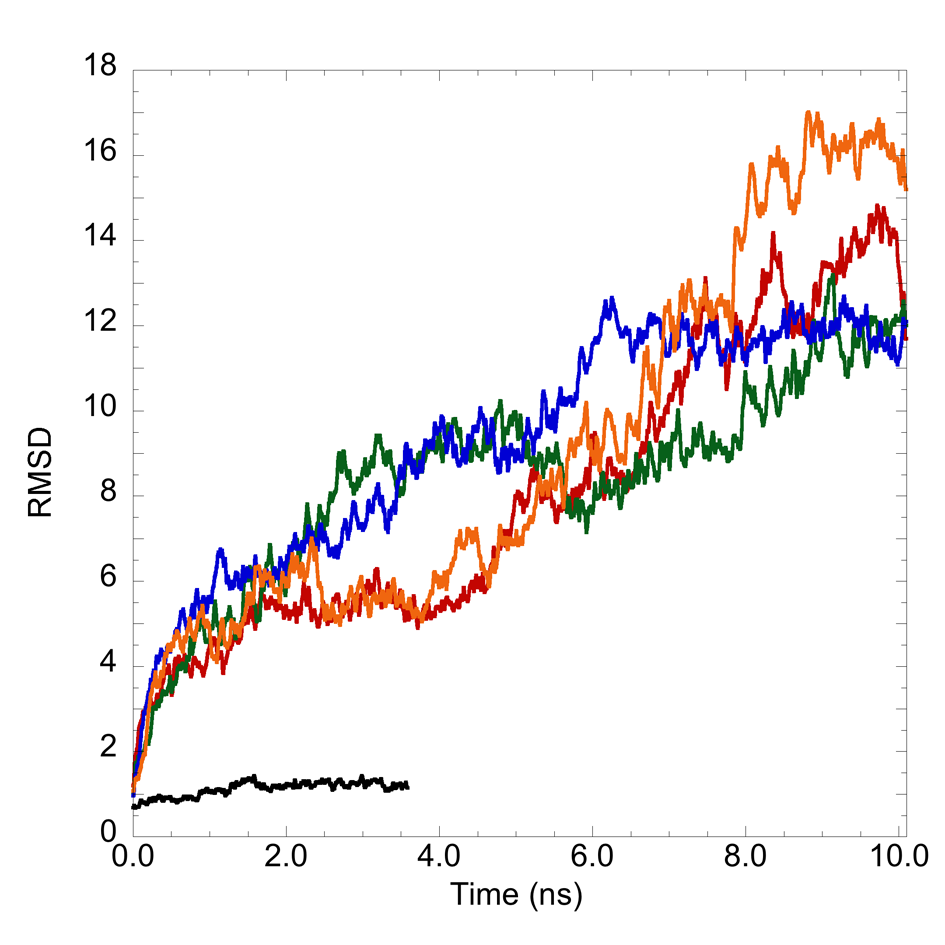

Supplement: Figure S2 — Cα RMSD for trypsin control and unfolding simulations (black, T298K; red, T500K1; green, T500K2; blue, T500K3; orange, T500K4). (0.26 MB TIF) [file pcbi.1000689.s005.tif]

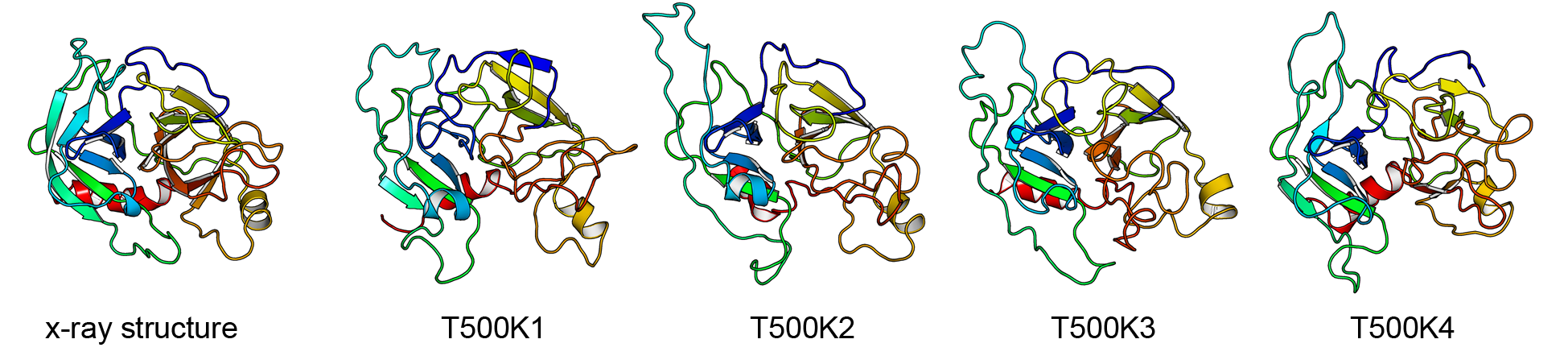

Supplement: Figure S3 — X-ray structure of trypsin and members of its unfolding TSE from each simulation. Some similarities are seen with αLP, particularly the maintenance of the β-sheet in the N-terminal domain and the C-terminal α-helix and the disruption of the domain interface both near the active site and at the “top” of the molecule as pictured. (0.57 MB TIF) [file pcbi.1000689.s006.tif]
